# Designed Ankyrin Repeat Proteins: A New Approach to Mimic Complex Antigens for Diagnostic Purposes?

**DOI:** 10.1371/journal.pone.0060688

**Published:** 2013-04-23

**Authors:** Stefanie Hausammann, Monique Vogel, Johanna A. Kremer Hovinga, Sebastien Lacroix-Desmazes, Beda M. Stadler, Michael P. Horn

**Affiliations:** 1 University Institute of Immunology, University of Bern, Inselspital, Bern, Switzerland; 2 Department of Hematology and Central Hematology Laboratory, University Hospital and University of Bern, Bern, Switzerland; 3 INSERM, UMR S 872, Les Cordeliers, Paris; 4 Université Pierre et Marie Curie-Paris, UMR S 872, Les Cordeliers, Paris; 5 Université Paris Descartes, UMR S 872, Les Cordeliers, Paris; Cordelier Research Center, INSERMU872-Team16, France

## Abstract

Inhibitory antibodies directed against coagulation factor VIII (FVIII) can be found in patients with acquired and congenital hemophilia A. Such FVIII-inhibiting antibodies are routinely detected by the functional Bethesda Assay. However, this assay has a low sensitivity and shows a high inter-laboratory variability. Another method to detect antibodies recognizing FVIII is ELISA, but this test does not allow the distinction between inhibitory and non-inhibitory antibodies. Therefore, we aimed at replacing the intricate antigen FVIII by Designed Ankyrin Repeat Proteins (DARPins) mimicking the epitopes of FVIII inhibitors. As a model we used the well-described inhibitory human monoclonal anti-FVIII antibody, Bo2C11, for the selection on DARPin libraries. Two DARPins were selected binding to the antigen-binding site of Bo2C11, which mimic thus a functional epitope on FVIII. These DARPins inhibited the binding of the antibody to its antigen and restored FVIII activity as determined in the Bethesda assay. Furthermore, the specific DARPins were able to recognize the target antibody in human plasma and could therefore be used to test for the presence of Bo2C11-like antibodies in a large set of hemophilia A patients. These data suggest, that our approach might be used to isolate epitopes from different sets of anti-FVIII antibodies in order to develop an ELISA-based screening assay allowing the distinction of inhibitory and non-inhibitory anti-FVIII antibodies according to their antibody signatures.

## Introduction

Coagulation factor VIII (FVIII) is a 300 kDa polypeptide acting as a cofactor in the intrinsic pathway of thrombin formation. It consists of a heavy chain (A1-a1-A2-a2-B) and a light chain (a3-A3-C1-C2), linked via a metal ion and circulates in the blood stabilized by von Willebrand factor (vWF). In hemophilia A (HA) patients deficiency or malfunction of FVIII causes severe bleeding diathesis [1]. Congenital HA, caused by mutations in the FVIII gene located on the X chromosome, occurs in one of 5000 males. Absent or non-functional FVIII is substituted with plasma-derived or recombinant FVIII. As a consequence of the treatment, 5–40% of HA patients develop allo-antibodies towards the therapeutic FVIII protein, depending on the type of FVIII gene mutation [2]. Such immune responses against infused FVIII represent serious complications of hemorrhage treatment. As inhibitors rapidly inactivate FVIII, treatment efficacy is dramatically reduced [3]. On the other hand, antibodies against FVIII were detected that bind to FVIII but do not interfere with its function. Such non-inhibitory anti-FVIII antibodies can be found in inhibitor positive and negative HA patients as well as in healthy controls [4], [5], [6]. The pathophysiological role of these non-inhibitory antibodies is unclear although they may increase clearance of circulating FVIII [7].

It is difficult to investigate the difference between inhibitory and non-inhibitory antibodies, as the antibody fractions cannot be separated and most approaches to measure anti-FVIII antibodies cannot distinguish between them. The Bethesda assay is the only method that selectively detects inhibitory antibodies but this test is time consuming, has a low sensitivity and despite different improvements shows a high inter-laboratory variation [8], which indicates the need for an alternative test.

We hypothesize that the epitope specificity of an antibody determines whether it is inhibitory or not, as antibodies binding to a functional site on FVIII can inhibit its pro-coagulant activity.

To discriminate between inhibitory and non-inhibitory antibodies, we aim at replacing the intricate and unstable antigen FVIII by artificial binding proteins describing the epitope signatures of anti-FVIII antibodies. As a proof-of-concept, we used the well-described human monoclonal anti-FVIII antibody Bo2C11 to select binders against its antigen-binding site. Bo2C11 is a high titer inhibitor derived from a congenital HA patient by EBV transformation of a memory B cell [9]. As most allogeneic FVIII inhibitors, Bo2C11 is an IgG4 antibody. It was shown to recognize a discontinuous epitope on the C2 domain of FVIII that is involved in the interaction of FVIII with vWF and phospholipids. This inhibitor therefore blocks FVIII activity by preventing the formation of the tenase complex.

Several approaches for epitope mapping of anti-FVIII antibodies have already been made. A murine antibody directed against the idiotype of a FVIII inhibitor was generated and peptide libraries were screened for anti-idiotypic binders to an inhibitor, to mention a few [10], [11]. It is not clear whether a murine antibody can mimic the epitope of a human antibody and short peptides have a rather small interaction site and limited potential to build three-dimensional structures. Therefore we used Designed Ankyrin Repeat Proteins (DARPins) as binding proteins for epitope mimicry. DARPins are based on natural ankyrin repeat proteins and were generated as described [12]. Briefly, the identification of conserved and variable residues on natural ankyrin repeat proteins led to the construction of a consensus repeat module with a theoretical variability of 7.2 × 10^7^. DARPin libraries containing 2 or 3 repeat modules resulting in 10^15^ and 10^23^ different binders were generated. The theoretical variability of the DARPin libraries is much higher than diversities of phage peptide libraries (10^9^), which increases the possibility to find highly specific binders. Due to their design, DARPin proteins can be generated *in vitro.* Additionally, DARPin proteins have a molecular size of 14 to 18 kDa providing a larger area of interaction than peptides, which makes them good candidates for epitope mimicry. DARPin binders recognizing various targets with high specificity and affinity have already been isolated [13], [14], [15]. In a previous study we showed that DARPins can be selected against the antigen-binding site of a murine monoclonal anti-IgE antibody [16]. Here we tested the ability of DARPins to mimic the epitope of the human monoclonal anti-FVIII antibody, Bo2C11, in order to assess their potential to replace the complex and unstable antigen FVIII. Using Ribosomal Display technology we successfully isolated DARPin binders that specifically recognize the binding site of the monoclonal anti-FVIII antibody, Bo2C11. We produced dimeric DARPins by joining two DARPins via a flexible protein linker. These constructs specifically blocked the binding of Bo2C11 to its natural antigen, FVIII and neutralized the antibody's inhibitory activity. Furthermore they could be used to detect Bo2C11 spiked into a healthy human plasma pool. Further studies can now be performed to explore the use of such molecules for epitope-specific screening of antibodies in patient blood samples in order to develop a screening test distinguishing inhibitory from non-inhibitory anti-FVIII antibodies.

## Materials and Methods

### 2.1. Recombinant FVIII, anti-FVIII antibodies and human plasma samples

Full-length recombinant FVIII (Kogenate® FS) was kindly provided by Bayer Healthcare. FVIII was reconstituted in H_2_O, dialyzed into Borate Buffer (100 mM H_3_BO_3_, 150 mM NaCl, 5 mM CaCl_2_ × 2H_2_O, pH 7.0) and stored frozen at −20°C in small aliquots until use.

The cell line producing a monoclonal IgG4κ antibody named Bo2C11, specific for human FVIII C2 domain and derived from a congenital HA patient [9] was a kind gift of Dr. M.G Jacquemin. The antibody was produced in serum-free medium (HL-1, Lonza, Basel Switzerland) and purified using a Protein G column (GE Healthcare, Chalfont St. Giles, UK). Fractions containing eluted antibodies were pooled, dialyzed into PBS (137 mM sodium chloride, 2.7 mM potassium chloride, 12 mM phosphate, pH 7.4) and concentrated using Vivaspin® columns (Sartorius Stedim Biotech GmbH, Göttingen, D). Protein concentrations were calculated from A_280_ using an extinction coefficient of 1.36. Antibodies were aliquoted and frozen at −20°C.

The study has been accepted by the local ethical committee (Kantonale Ethikkommission Bern (KEK), CH-3010 Bern). All patients and healthy plasma donors in the study signed a written informed consent.

### 2.2. Vectors and libraries

The vectors pRDV (GenBank accession no. AY327136), used for ribosome display, pQi-bi-2-2, needed to generate dimeric DARPins, as well as the expression vector pMPAG6 were received from Molecular Partners AG (Schlieren, Switzerland). For expression of monomeric DARPins the vector pMPAG6 was used, a modified vector analogous to the commercially available backbone pQE30 (Qiagen, Hilden, Germany), which contains a His_6_-Tag sequence. For the expression of DARPins we used *E.coli* XL-1 Blue (Stratagene, San Diego, CA). To obtain dimeric constructs two DARPin DNA fragments were cloned into pQi-bi-2-2 containing a [Gly_4_-Ser]_4_ linker located between BamHI/HindIII and BglII/BsaI cloning sites that allow site-directed DARPin insertion.

Two DARPin DNA libraries, coding for DARPins with 2 (N2C) or 3 (N3C) repeat modules, were obtained from Molecular Partners AG. Details on library construction have been published elsewhere [17].

### 2.3. *In vitro* selection and DARPin expression

The selection of DARPins was performed using Ribosome Display as described earlier [18], [19], [20]. We used both N2C and N3C DARPin libraries to find binders to the variable region of the human monoclonal anti-FVIII antibody Bo2C11 (IgG4). Two selection rounds were performed on the target antibody, including a pre-adsorption step on PBS containing 0.15% Casein (PBS-C) to remove non-specific binders. In the third round a pre-adsorption step on two different IgG4 antibodies of non-relevant specificity was included to remove binders against the constant region. The number of cycles for the PCR on cDNA was reduced from 45 to 35 and 30 from panning round 1 to 3, respectively.

Amplified DARPin sequences of the third panning round were cloned into pMPAG6 vector for DARPin expression in *E.coli* XL1-Blue cells. Crude extracts of different single DARPin clones were produced for ELISA specificity screening as described earlier [21]. Briefly, overnight cultures were grown in selection medium (LB, containing 100 µg/ml ampicillin) until an optical density of 0.6 at 600 nm was reached, then protein expression was induced with 1 mM isopropyl-β,D-thiogalactopyranoside (IPTG) (AppliChem, Darmstadt, Germany) for 3 h. Cells were harvested by centrifugation (20 min 3400 g), lysed with B-PER® (Thermo Fisher Scientific, Waltham, MA, USA) and diluted in TBS500 (50 mM Tris-HCl pH 8.0, and 500 mM NaCl) containing a protease inhibitor cocktail (Roche, Basel, Switzerland) to give 1 ml clarified crude extract after centrifugation.

### 2.4. Analysis of DARPin binding properties to human IgG

To analyze DARPin binding specificity crude extracts of 96 different N2C single clones (see 2.3) were tested on the anti-FVIII antibody, Bo2C11. 33 nM of the target antibody diluted in PBS were immobilized on a Corning® 96-well Microplate (microplate) (Corning Incorporated, NY, USA) overnight at 4°C. Microplates were washed twice with PBS and blocked with 150 µl PBS-C for 2 h at 37°C. Subsequently, 50 µl of crude extracts diluted 1∶10 in PBS-C were incubated for 1 h at 37°C, then plates were washed 4 x with PBS containing 0.01% Tween-20 (PBS-T) and 4 x with PBS. Binding of DARPin proteins was revealed with a biotinylated anti-polyhistidine (anti-His_6_) antibody (R&D Systems, Minneapolis, MN, USA; 1∶1000) followed by peroxidase-labeled streptavidin (Dako, Glostrup, Denmark; 1∶1000) after washing as described above. DARPin binding was detected by 3,3′, 5,5′-tetramethylbenzidine (TMB, Fluka, St. Louis, MO, USA) and color reaction was stopped after 5 min with 1 M H_2_SO_4_. Optical density was read at 450 nm in a standard ELISA reader (BIO-TEK EL808, BioTek, Bad Friedrichshall, Germany). Positive clones were retested at the same dilution, including a human IgG4 antibody with non-relevant specificity and the blocking agent, PBS-C, as negative controls. Bound DARPins were detected with a murine monoclonal anti-RGS His_6_ antibody (1∶1000; Qiagen), followed by a horseradish-peroxidase-conjugated goat anti-mouse IgG (1∶5000; Jackson ImmunoResearch, West Grove, PA, USA). DARPin binding was visualized with TMB as described above.

For further experiments DARPin proteins were purified over a TALON™ metal-affinity chromatography column according to the manufacturer's instructions (Clontech, CA, USA). The purity and integrity of DARPins were confirmed by SDS PAGE and Western blotting (Figure S1).

### 2.5. Sequencing of DARPins

From the overnight cultures, plasmid DNA was extracted using a Maxi prep kit (Qiagen). DARPin-encoding DNAs were sequenced using BigDye Terminator v3.1 Cycle sequencing kit. PCR reactions were purified with BigDye XTerminator Purification kit, read on the ABI 3130X Genetic Analyzer and analyzed with Sequencing Analysis software v5.2 (all from Applied Biosystems, CA, USA).

### 2.6. Generation and characterization of dimeric DARPins

Two monomeric DARPins, eBo01 and eBo38, were cloned into pQi-bi-2-2 vector. The first DARPin sequence was digested with *Bam*HI and *Hind*III (Roche, Basel, Switzerland) and ligated using T4 ligase (Invitrogen, Carlsbad, CA, USA). The second DARPin was introduced downstream of the first fragment using *Bgl*II and *Bsa*I (New England Biolabs, MA, USA) restriction sites. All four combinations of the two DARPins were generated and constructs were produced in *E.coli* XL-1 Blue as described in 2.4 in large expression cultures (250 ml). Cells were lysed with French Press (15000PSI) (Thermo Fisher Scientific, Waltham, MA, USA) and dimeric DARPins were purified on TALON® resin. The purity and integrity of DARPins were confirmed by SDS PAGE and Western blotting (Figure S1). Binding of purified mono- and dimeric DARPins to Bo2C11 and human IgG subclasses was compared. Microplates were coated with 13.3 nM antibodies, washed and blocked as above. 150 nM of monomeric DARPins (eBo01 and eBo38) and 4 nM of dimeric DARPins (eBo01-38 and eBo38-38) were incubated for 1 h at 37°C. After a washing step DARPin binding was visualized by an anti-RGS His_6_ antibody and a peroxidase labeled anti-mouse IgG antibody as described above.

### 2.7. Affinity measurements

Binding strength of purified DARPins was analyzed by surface plasmon resonance analysis on a Biacore X100 instrument. HBS-EP^+^ (10 mM HEPES, 150 mM NaCl, 3 mM EDTA, pH 7.4 containing 0.05% Surfactant P20) was used as running buffer (flow rate 30 µl/min). 2100 Response Units of Bo2C11 were immobilized on one of the flow cells of a CM5 sensor chip, whereas the other flow cell remained uncoated and served as a reference. To assess the association rates, samples were injected for 3 min at different concentrations (1 nM to 40 nM for monomeric, 0.1 nM to 6 nM for dimeric DARPins) and the dissociation rates were measured for another 3 min. A buffer control was measured and subtracted from the sensorgram of each sample and binding parameters were determined using Biacore X100 evaluation software 2.0 (all from GE Healthcare).

### 2.8. Competition between DARPins and FVIII for Bo2C11

In a first step the concentration of Bo2C11 giving a 50% maximal signal (EC_50_) on immobilized FVIII was determined. For this purpose FVIII (6.6 nM in PBS) was coated on a microplate followed by a 2 h blocking step using PBS-C. Plates were washed as described above. Bo2C11 was serially diluted 1∶3 starting at a concentration of 60 nM and the amount of antibody bound to FVIII was determined using a horseradish-peroxidase-conjugated sheep anti-human IgG antibody (The Binding Site, Birmingham, UK). The determined concentration of Bo2C11 for EC_50_ (2.66 nM) was used in the inhibition assay.

In the inhibition ELISA 5.33 nM (2x EC_50_) of Bo2C11 were mixed 1∶1 with different concentrations of dimeric DARPins in the range of 10^−3^ - 10^3^ molar excess. These mixtures were pre-incubated for 1 h at room temperature and then added to FVIII coated wells. Plates were incubated at 37°C for 1 hour and washed as above. Residual Bo2C11 binding to FVIII was detected by a horseradish-peroxidase-conjugated sheep anti-human IgG antibody (The Binding Site) and developed with TMB as described above.

### 2.9. Bethesda Assay

First, the concentration of Bo2C11 inhibiting FVIII pro-coagulant activity by 50%, defined as 1 Bethesda Unit (BU) was determined empirically, as batch-to-batch variation occurs. Bo2C11 was diluted in veronal acetate buffer containing 1 mg/ml bovine serum albumin and mixed 1∶1 with commercial normal plasma exhibiting known FVIII activity. As a reference, FVIII containing standard plasma was mixed 1∶1 with FVIII deficient plasma, which results in a theoretical FVIII activity of 50%. Samples were incubated for 2 h at 37°C and coagulation was measured on a Behring Coagulation System (all from Siemens Healthcare Diagnostics, Deerfield, USA). Residual FVIII activity in percent was calculated relatively to the reference value.

According to this first experiment, 3 nM of Bo2C11 (1BU) were used for the neutralization assay. Dimeric DARPins were pre-incubated with Bo2C11 at molar ratios ranging from 10^−3^ to 10^3^ for 1 h at room temperature. The mixture was diluted 1∶2 with normal plasma, coagulation was measured and residual FVIII activity was calculated as above.

### 2.10. Detection of Bo2C11 in human plasma

The ability of DARPins to recognize Bo2C11 in human plasma was analyzed by ELISA and a catching assay. For the analysis by ELISA, one representative of Bo2C11-specific DARPins (eBo01-38) and a control DARPin were immobilized at 2 µg/ml on a microplate overnight at 4°C. Microplates were washed and blocked as above. Bo2C11 was spiked at different concentrations into a plasma pool of 4 healthy controls (diluted 1∶100 in PBS-C) and the mixtures were incubated for 2 h at 37°C on the microplate. After washing, Bo2C11 binding to the DARPins was detected using a peroxidase-labeled anti-human IgG antibody as above. TMB was used for color development and optical density was determined as above. The detection limit was defined as the mean value of the diluted plasma pool without Bo2C11 + 2 SD. For the catching assay three concentrations (1 µg, 0.1 µg and 0.01 µg) of a murine anti-His_6_ antibody were coated on a nitrocellulose membrane. Commercial human plasma diluted 1∶50 in PBS-C was spiked with 2 µg/ml of either Bo2C11 or a control human IgG, or nothing. Samples were then mixed with equimolar (13.3 nM) amounts of either eBo38-38 or a control DARPin or without any DARPin for control purposes. Bound human IgGs were detected using a peroxidase-conjugated sheep anti-human IgG antibody (The binding Site). The presence of the coating antibody was confirmed with a peroxidase-conjugated goat anti-mouse IgG antibody (Jackson ImmunoResearch). Nitrocellulose strips were developed using a 0.05% 4-chloro 1-naphthol solution and scanned.

## Results

### 3.1. Selection of Bo2C11 binders

To test whether DARPins are able to mimic the relevant epitopes of intricate antigens we used the well-described human anti-FVIII antibody Bo2C11 for the isolation of specific DARPin. Thus, DARPin libraries consisting of two (N2C) or three (N3C) randomly associated variable ankyrin repeat modules were screened on Bo2C11. Three selection rounds were performed as described in the Materials and Methods section.

**Figure 1:**
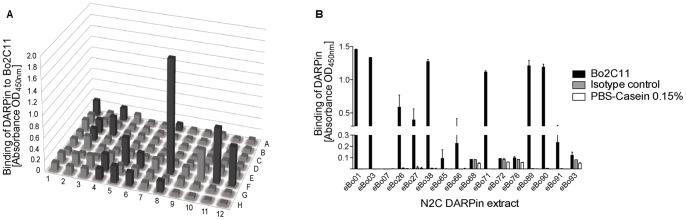
Screening of DARPin clones after third selection round. A) Crude extracts of 96 individual DARPin clones were tested for binding to the target antibody, Bo2C11. Extracts were diluted 1∶10 and binding of DARPins was revealed using a murine anti-His_6_ antibody and a peroxidase-labeled anti-mouse IgG antibody. Clones 1–12 are shown in row A, clones 13–24 in row B and so on. Clones chosen for retesting are highlighted in dark grey. B) Re-testing of positive clones. The highlighted clones depicted in Fig.1A were again tested on Bo2C11 (black bars), an isotype control antibody (grey bars) and the blocking agent (white bars) diluted 1∶10. DARPin binding was revealed with a murine anti-His_6_ antibody and a horseradish-peroxidase labeled goat anti-mouse antibody.

**Figure 2:**
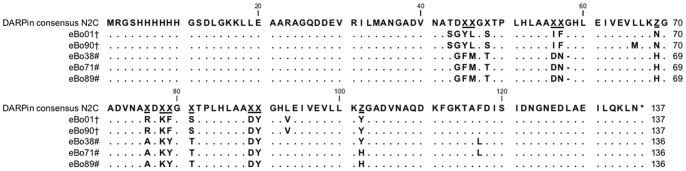
Sequence analysis of Bo2C11–specific DARPin clones. Plasmid DNA of positive clones was sequenced and aligned with the N2C DARPin consensus sequence. In the consensus module position X allows any amino acid except Cys, Pro or Gly and Z can be either His, Asp or Tyr. Based on the sequence homologies, DARPins were grouped to group 1(†) or group 2 (#), respectively. Identical residues are displayed as dots.

Crude extracts of 96 individual DARPin clones (A #1–12; B #13–24, C #25–36, D #37–48, E #49–60, F #61–72, G #73–84, H #85–96) were screened for their binding to the target antibody, Bo2C11 in ELISA (Fig. 1A). Sixteen clones with a high signal on Bo2C11 (highlighted in dark grey) were selected for re-testing on Bo2C11 and an isotype control. Eight of the 16 N2C DARPin clones recognized Bo2C11, whereas only low reactivity to control proteins was observed (Fig. 1B). From the N3C DARPin pool, none of the clones was specific for the target antibody (data not shown). The six N2C clones with the highest signal-to-noise ratio (eBo01, eBo03, eBo38, eBo71, eBo89 and eBo90) were sequenced and aligned to test diversity of binders (Fig. 2). We were unable to sequence clone eBo03 and therefore this clone was excluded from further analyses (data not shown). Based on common framework mutations the clones were subdivided into 2 groups. Group 1 consisted of eBo01 and eBo90 and group 2 of eBo38, eBo71 and eBo89. The sequence homology within the groups was 99.2% (group 1) and 98.5% (group 2), respectively, and between the two groups 90.5%. Together these results indicated that one or two major epitopes can be isolated by a monoclonal antibody. Finally, four DARPins, two of each group (eBo01 and eBo90 from group 1; eBo38 and eBo89 from group 2) were selected for further experiments.

### 3.2. Affinity and binding specificity of DARPins

For further characterization, the selected DARPin clones were produced in large amounts in bacteria and purified on metal affinity columns by interaction with their His_6_-tag. The purity and integrity of the proteins were confirmed by SDS PAGE and Western blotting (Fig. S1). The affinities of the four clones, as determined by surface plasmon resonance analysis were comparable and in the low nanomolar range (1.36 × 10^−8^, 4.27 ×10^−8^, 2.53 ×10^−8^ and 3.19 ×10^−8^ for eBo01, eBo90, eBo38 and eBo89, respectively; Table 1B). Based on these similarities we chose DARPins eBo01 and eBo38, one member of each group, for further experiments.

**Table 1:** Affinities of DARPins and FVIII to Bo2C11.

	Binder Type	Designation	*k_a_* [M^−1^s^−1^]	*k_d_* [s^−1^]	*K_D_* [M]
**A.**	Coagulation factor VIII^a^	FVIII	7.40 × 10^5^	1.6 × 10^−4^	1.4 × 10^−11^
**B.**	Monomeric DARPins				
	Group 1	eBo01	2.53 × 10^7^	0.3457	1.36 × 10^−8^
		eBo90	1.33 × 10^6^	0.0626	4.71 × 10^−8^
	Group 2	eBo89	1.28 × 10^7^	0.4072	3.19 × 10^−8^
		eBo38	1.45 × 10^7^	0.3694	2.53 × 10^−8^
**C.**	Dimeric DARPin constructs				
		eBo01-01	1.01× 10^6^	1.14× 10^−4^	1.13 × 10^−10^
		eBo38-38	1.99 × 10^6^	6.65 × 10^−4^	3.34 × 10^−12^
		eBo01-38	6.54 × 10^6^	1.36 × 10^−4^	2.07 × 10^−11^
		eBo38-01	6.49 × 10^6^	2.01 × 10^−4^	3.10 × 10^−11^

a Published values [9].

**Figure 3:**
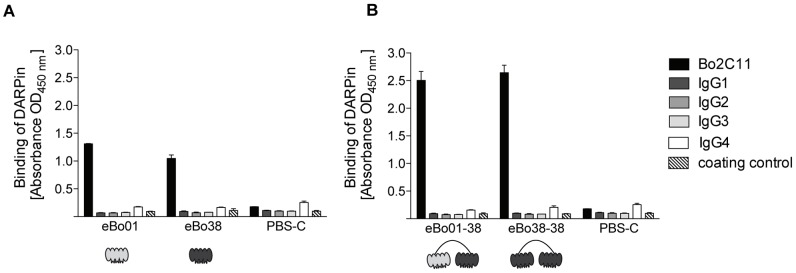
Specificity analysis of selected DARPin clones by ELISA. Purified DARPin proteins were tested for binding to the human monoclonal anti-FVIII antibody Bo2C11 (black bars), human IgG subclass antibodies of non-relevant specificity (IgG1 dark grey bars, IgG2 grey bars, IgG3 light grey bars, IgG4 white bars) and the coating control (PBS containing 0.15% Casein) (striped bars) in ELISA. DARPin binding was revealed using a monoclonal mouse antibody directed against the N-terminal RGS-His_6_-tag of DARPins and a horseradish-peroxidase-labeled goat anti-mouse antibody. A) shows the binding of 150 nM of monomeric DARPins eBo01 and eBo38. B) shows the binding of 4 nM of dimeric DARPins eBo01-38 and eBo38-38. As control in A) and B), PBS-C was incubated without DARPins on the human antibodies.

To investigate whether DARPins specifically recognized the variable part of the monoclonal antibody Bo2C11, purified DARPin proteins were tested against human IgG subclass antibodies of non-relevant specificities and against the blocking agent (Fig. 3A). DARPins eBo01 and eBo38 specifically bound to Bo2C11, while no reaction to the IgG subclasses or the blocking agent was observed. Binding of eBo01 to the target antibody was in the same range as that of eBo38, confirming the results obtained in surface plasmon resonance analysis. The signal of both DARPins on IgG4 was slightly increased compared to the other IgG subclass antibodies but did not exceed the background value of uncoated, blocked wells. Together these data indicate that DARPins eBo01 and eBo38 do not react with the constant region of IgG subclass antibodies and are specific for the binding sites of Bo2C11.

### 3.3. Generation and characterization of dimeric DARPins

We previously observed that linking two DARPins allowed to efficiently immobilize DARPins on a solid phase without affecting specificity (data not published) and also could cause an increase in binding strength due to an avidity effect. DARPins eBo01 and eBo38 were used to generate dimeric DARPin constructs with both orientations, expressed in *E.coli* XL-1 Blue and purified from extracts as described in Materials and Methods. The purity and integrity of the proteins were confirmed by SDS PAGE and Western blotting (Fig. S1).

Surface plasmon resonance analysis revealed that binding of the dimeric DARPins was increased by approximately thousand-fold over their monomeric equivalents, resulting in affinities in the low picomolar range (Table 1C). Importantly, affinities of dimeric DARPins to Bo2C11 were in the same range as the binding strength between FVIII and Bo2C11 (Table 1A). The combination eBo01-01 showed the lowest affinity to Bo2C11 and was left out for further experiments. In contrast to others [22], we did not observe an effect of the orientation of DARPins eBo01 and eBo38 on binding strength.

To verify the specificities of dimeric DARPins their binding to Bo2C11 and different human IgG subclass antibodies was analyzed by ELISA (Fig. 3). Both monomeric DARPins eBo01 and eBo38 (Fig. 3A) and dimeric DARPins eBo01-38 and eBo38-38 (Fig. 3B) were tested at different concentrations on Bo2C11 and human IgG subclass control antibodies. An increased binding to Bo2C11 of both dimeric DARPins compared to monomeric DARPins was observed, even when using about 40 times less dimeric over monomeric proteins. No binding to any of the human IgG subclass antibodies was observed. We did not observe any difference in reactivity against Bo2C11 between the two constructs, indicating that the increased binding is mainly due to an avidity effect.

### 3.4. DARPins inhibit Bo2C11 binding to FVIII

**Figure 4:**
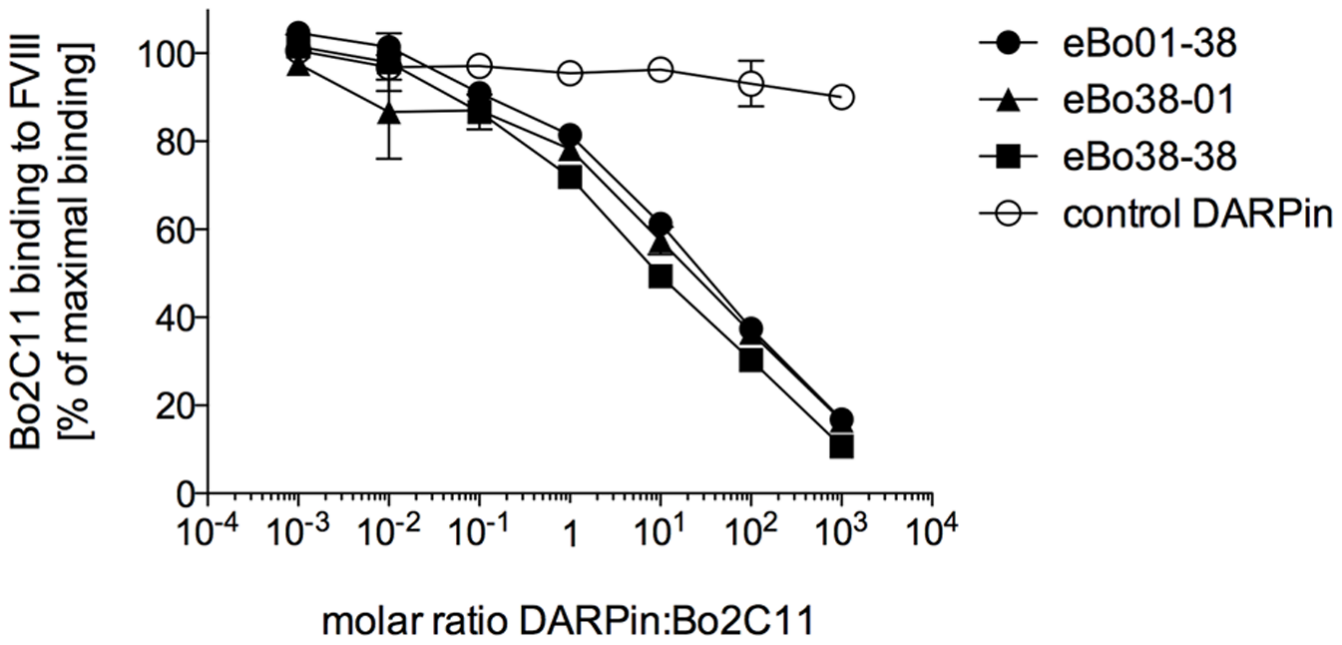
Bo2C11 binding to FVIII in presence of dimeric anti-FVIII antibody-specific DARPins. Inhibition of anti-FVIII antibody (Bo2C11) binding was performed at the EC_50_ value of Bo2C11 on FVIII. 800 ng/ml Bo2C11 was mixed 1∶1 with increasing concentrations of eBo01-38 (black circles), eBo38-01 (black triangles), eBo38-38 (black squares) or a dimeric DARPin of non-relevant specificity (open circles). The mixtures were pre-incubated at room temperature for 30 min and then added to FVIII-coated wells. The amount of Bo2C11 binding to FVIII was revealed with a horseradish-peroxidase-labeled anti-human IgG antibody. The results were normalized to 800 ng/ml Bo2C11 mixed 1∶1 with buffer on FVIII. Data represent mean and standard deviation of duplicates. Shown is one representative of 3 individual experiments.

To investigate whether the Bo2C11-specific (eBo) DARPins recognize specifically the binding site of the monoclonal anti-FVIII antibody Bo2C11, an inhibition ELISA was performed (Fig. 4). A final concentration of 400 ng/ml or 2.66 nM, corresponding to the EC_50_ value of Bo2C11, was incubated with different amounts of dimeric eBo DARPins ranging in molar ratio from 10^−3^ to 10^3^. The residual amount of Bo2C11 that bound to FVIII coated on the solid phase was assessed. All three Bo2C11-specific DARPin constructs inhibited the binding of Bo2C11 to FVIII in a dose-dependent manner, whereas a dimeric DARPin of non-relevant specificity did not have any effect. The highest inhibition of 89% was observed with DARPin eBo38-38 using a thousand-fold molar excess of DARPin over Bo2C11. Inhibition of the binding of Bo2C11 to FVIII by eBo DARPins indicates that they are directed against the antigen-binding site of Bo2C11.

### 3.5. Neutralization of inhibitory activity of Bo2C11

**Figure 5:**
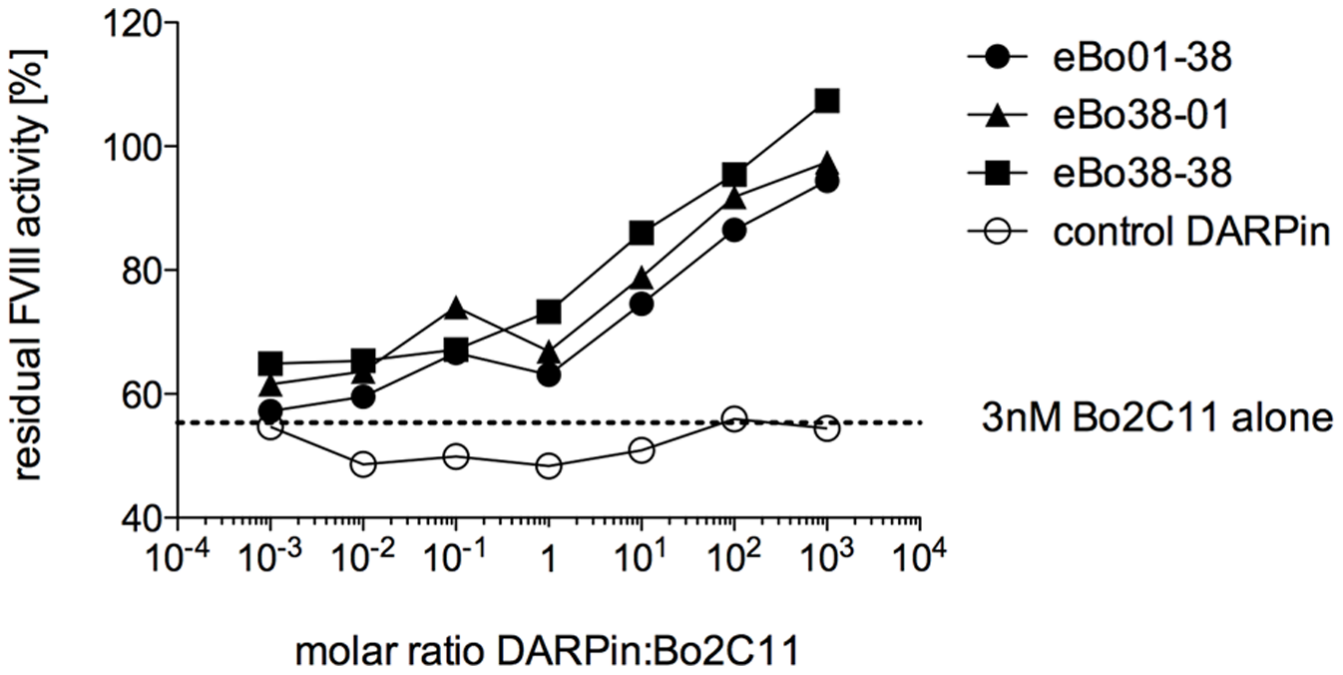
Anti-Bo2C11 DARPins neutralize inhibitory activity of Bo2C11. Different concentrations of three dimeric DARPins recognizing the monoclonal anti-FVIII antibody Bo2C11 (eBo01-38: closed circles; eBo38-01: closed triangles; eBo38-38: closed squares) or a dimeric control DARPin (open circles) were mixed 1∶1 with 6 nM of Bo2C1, added to human standard plasma and analyzed in the modified Bethesda assay. 3 nM of the monoclonal anti-FVIII antibody Bo2C11 corresponded to 1 Bethesda Unit (BU) (dotted line). Values were normalized to standard plasma and expressed as residual FVIII activity.

As antibody binding is required for the inhibition of FVIII activity, we investigated whether the eBo DARPins could restore the pro-coagulant activity of FVIII in presence of Bo2C11 using a Bethesda Assay. Bo2C11 at a concentration of 450 ng/ml or 3 nM, corresponding to a titer of 1 BU/ml in our system, was mixed with different concentrations of the three dimeric DARPins or a control DARPin, ranging in molar ratios from 10^−3^ to 10^3^ (Fig. 5). All Bo2C11-specific DARPins restored pro-coagulant activity of FVIII in a dose-dependent manner, whereas a control DARPin had no effect. A thousand-fold molar excess of Bo2C11-specific DARPins resulted in an increase of FVIII activity to 99.7% in average (SD = 6.7%). The maximal effect was achieved with the dimeric DARPin eBo38-38 in agreement with ELISA binding data.

Together, these data indicate that anti-Bo2C11 DARPins represent the epitope of Bo2C11.

### 3.6. Detection of Bo2C11-like antibodies in human plasma

**Figure 6:**
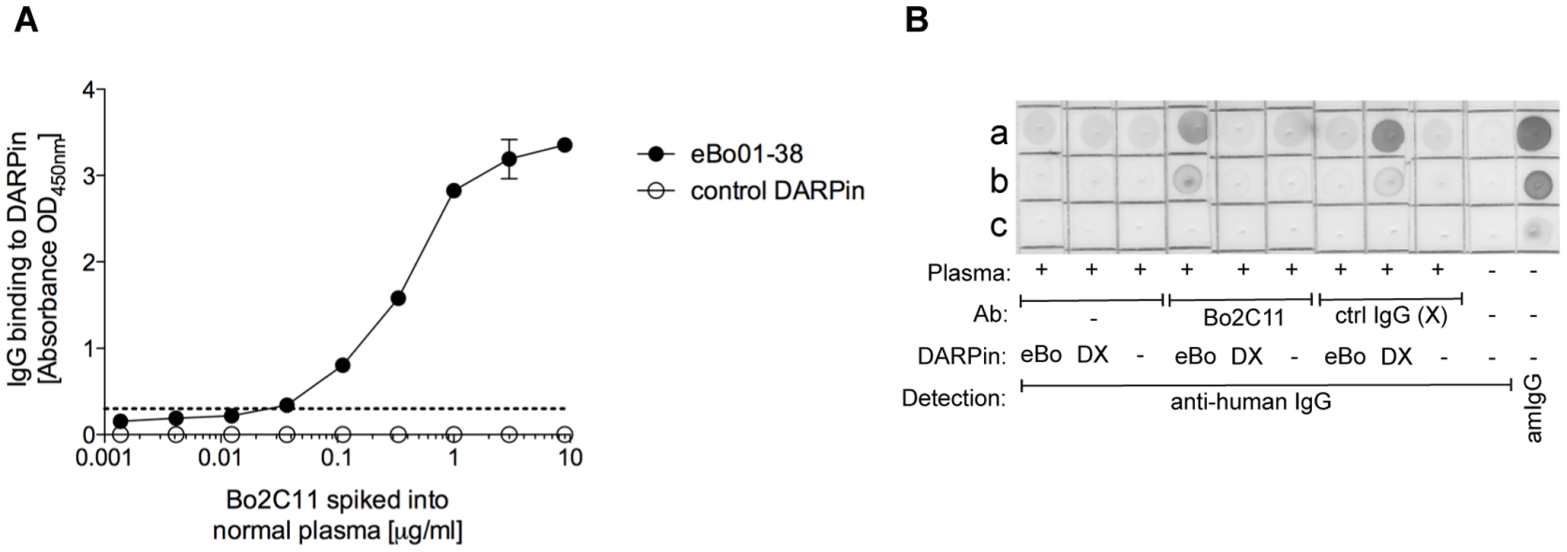
Dimeric DARPins recognize Bo2C11 spiked in healthy plasma. A) Detection of Bo2C11 by DARPins in ELISA. DARPin eBo01-38 (closed circles) or a dimeric control DARPin (open circles) were coated at 69 nM in PBS on a microtiter plate. 3-fold serial dilutions (60 nM – 9.3 pM) of Bo2C11 in a plasma-pool from healthy donors (1∶100 in PBS containing 0.15% casein and 0.1% tween-20) were incubated on immobilized DARPins. Bo2C11-binding to DARPins was detected using a horseradish-peroxidase-labeled sheep anti-human IgG antibody. The dotted line represents the detection limit of Bo2C11 in plasma on DARPin eBo01-38 (mean of diluted plasma + 2 SD). B) Catching of Bo2C11 from human plasma with DARPins. An anti-His_6_ antibody was dotted at 1 µg (a), 0.1 µg (b) or 0.01 µg (c) to a membrane, respectively. Human standard plasma diluted 1∶50 alone or spiked with either 2 µg/ml Bo2C11 (Bo2C11) or a control human IgG (X) was mixed with eBo38-38 (eBo), a DARPin specific for the control IgG (DX) or nothing (-). Complexes of human IgG and DARPins were detected using a peroxidase-labeled murine anti-human IgG antibody. As controls the detection antibody was incubated on the membrane alone and the presence of the anti-His_6_ antibody on the membrane was confirmed with a peroxidase-labeled sheep anti-murine IgG antibody (amIgG).

As we intend to use the eBo DARPins to describe the signature of anti-FVIII antibodies, we tested their ability to detect Bo2C11 in human plasma using two different immunoassays. In the ELISA assay, a plasma pool of healthy donors was spiked with different concentrations of Bo2C11 (Fig. 6A). eBo01-38 as a representative of the eBo DARPins was able to detect Bo2C11 in human plasma in a dose-dependent manner, whereas no Bo2C11 binding to the control DARPin was observed. The sensitivity of the assay was calculated to be 33 ng/ml.

The binding specificity of eBo DARPins to Bo2C11 was confirmed in a catching assay (Fig. 6B). eBo38-38 but not a control DARPin was able to precipitate Bo2C11 spiked into human standard plasma on a membrane coated with an anti-His_6_ antibody. eBo38-38 did not precipitate a human control IgG spiked into the plasma at the same concentration, whereas the DARPin specific for the control IgG did. We observed some background of the plasma on the anti-His_6_ antibody, which was independent of the presence of DARPins and could be interpreted as anti-mouse IgG antibodies present in the plasma.

These data suggest that eBo DARPins might be used to detect Bo2C11-like antibodies in human plasma samples and thus DARPin binding patterns could be used to replace complex antigens for the development of ELISA- or Luminex-based diagnostic tools.

## Discussion

FVIII inhibitors seriously complicate HA treatment and are routinely detected using the Bethesda Assay. However, this assay shows limited sensitivity and high inter-laboratory variation while being labor intensive [8]. In addition to inhibitory anti-FVIII antibodies, non-inhibitory anti-FVIII antibodies are present in human plasma that are detected in ELISA using recombinant FVIII [6]. This assay, though, cannot distinguish between inhibitory and non-inhibitory antibodies. Therefore we aim at developing an ELISA assay based on artificial binding proteins describing the antibody signatures of inhibitory anti-FVIII antibodies. In a previous study we showed that DARPins can be selected against the antigen-binding site of a murine anti-IgE antibody [16]. Here, we show the proof-of-concept for FVIII epitope mimicry with DARPins using the inhibitory human monoclonal anti-FVIII antibody, Bo2C11.

DARPins selected against idiotypic determinants of Bo2C11 not only prevented the binding of Bo2C11 to FVIII, but were also able to neutralize its inhibitory activity in a functional test. Our experiments show that DARPins can be used to mimic relevant epitopes of an intricate antigen. Several approaches for epitope mapping of anti-FVIII antibodies were already tested. Screening of anti-FVIII antibody specificity on FVIII heavy and light chains [23] does not allow to differentiate between inhibitory and non-inhibitory anti-FVIII antibodies as no differences in domain specificity was observed between the two antibody groups [24]. Random peptide libraries [11] or peptide sequences derived from FVIII [25] were used to mimic the epitope of several human and mouse monoclonal anti-FVIII antibodies. Although sequence relevance is ensured when peptides are derived from FVIII, these peptides might not represent relevant epitopes, as they are linear. Additionally, discontinuous epitopes are lost with this approach and new (non-relevant) epitopes might be generated from sequences hidden in native FVIII. By using random peptide libraries for epitope mimicry, Villard and coworkers were able to avoid the generation of non-relevant and the loss of discontinuous epitopes [11]. They found binders representing the epitope of Bo2C11 in constrained libraries but not in linear peptide libraries, indicating that a three-dimensional structure better mimics the epitope of this antibody. The fact that the rigid backbone and the flexible loops of DARPins can be involved in target binding indicates that their potential to represent three-dimensional structures is increased over that of small peptides. The monomeric DARPins were able to neutralize the binding of Bo2C11 to FVIII comparable to the constrained peptides (data not shown), whereas dimeric DARPins have an approximately hundred times higher neutralizing activity. Both the peptides and DARPins do not show any amino acid sequence identity with the part of C2 domain that was shown to be involved in Bo2C11 binding (data not shown) [26]. Therefore, it is possible that the DARPins mimic surface properties of C2 without sequence homology, as was also suggested for the constrained peptides.

The selected DARPins were able to detect Bo2C11 in human plasma and therefore could be used to set up a new assay to screen for Bo2C11-like antibodies in blood samples. To evaluate the usefulness of the DARPins for such a diagnostic test we screened for Bo2C11-like antibodies in 12 sera from HA patients with anti-C2 antibodies. We did not find sera reacting stronger with eBo01-38 compared to a control DARPin (data not shown). As we are looking for one unique epitope reacting with the monoclonal anti-FVIII inhibitor Bo2C11, it is likely that no Bo2C11-like antibodies are present in this small cohort of HA patients analyzed. Another possibility is that the sensitivity of the assay is too low to detect low frequencies of Bo2C11-like antibodies in the samples. The assay might be optimized in order to increase its sensitivity and therefore the probability to identify low frequency antibodies.

In summary, we have described two DARPin clones that recognize one dominant idiotypic determinant on Bo2C11. These DARPins were able to compete with the cognate antigen of Bo2C11, FVIII, for binding, suggesting that similar epitopes are present on the DARPin and on FVIII. These data will allow us to apply this method for the isolation of epitopes from polyclonal anti-FVIII antibody mixtures thereby describing the anti-FVIII antibody signatures of HA patients. These signatures will provide insight into the molecular mechanisms of the antibody responses against FVIII and will serve to generate an array-based assay for the assessment of the reactivity patterns of anti-FVIII antibodies in HA patients and such assay may provide a tool to distinguish inhibitory from non-inhibitory anti-FVIII antibodies in the future.

## Supporting Information

Figure S1 Purity and integrity of DARPins analyzed by SDS PAGE and Westernblot. Monovalent and divalent DARPins used in this study were loaded at 30pmoles per lane on a 12% acrylamide gel. Proteins were stained with silver ions (A) or blotted to a nitrocellulose membrane, stained with a murine anti-His_6_ antibody (Qiagen) followed by a peroxidase labeled anti-mouse IgG antibody (Jackson ImmunoResearch) and developed with chemiluminescence (B). 1, eBo01; 2, eBo38; 3, eBo89; 4, eBo90; 5, eBo01-01, 6, eBo01-38; 7, eBo38-01; 8, eBo38-38. Staining of eBo01-01 with silver ions bleached, the presence of protein was confirmed with coomassie-staining (not shown). A small degree of polymerization of both monomeric and dimeric DARPins is observed, as is usually the case. Also some degradation is visible and probably a small contamination of dimeric DARPins with monomeric ones (lines 6–8).
(TIF)

## References

[1] Lenting P, van Mourik J, Mertens K (1998) The life cycle of coagulation factor VIII in view of its structure and function. Blood 92: 3983–3996. 9834200

[2] Ghosh K, Shetty S (2009) Immune response to FVIII in hemophilia A: an overview of risk factors. Clin Rev Allergy Immunol 37: 58–66. 19148784 10.1007/s12016-009-8118-1

[3] Gringeri A, Mantovani LG, Scalone L, Mannucci PM (2003) Cost of care and quality of life for patients with hemophilia complicated by inhibitors: the COCIS Study Group. Blood 102: 2358–2363. 12816859 10.1182/blood-2003-03-0941

[4] Coutinho A, Kazatchkine MD, Avrameas S (1995) Natural autoantibodies. Curr Opin Immunol 7: 812–818. 8679125 10.1016/0952-7915(95)80053-0

[5] Irigoyen MB, Primiani L, Felippo M, Candela M, Bianco RP, et al (2011) A flow cytometry evaluation of anti-FVIII antibodies: correlation with ELISA and Bethesda assay. Haemophilia 17: 267–274. 21070488 10.1111/j.1365-2516.2010.02406.x

[6] Klintman J, Hillarp A, Donfield S, Berntorp E, Astermark J (2012) Antibody formation and specificity in Bethesda-negative brother pairs with haemophilia A. Haemophilia. 10.1111/j.1365-2516.2012.02903.x 22762454

[7] Dazzi F, Tison T, Vianello F, Radossi P, Zerbinati P, et al (1996) High incidence of anti-FVIII antibodies against non-coagulant epitopes in haemophilia A patients: a possible role for the half-life of transfused FVIII. Br J Haematol 93: 688–693. 8652395 10.1046/j.1365-2141.1996.d01-1705.x

[8] Verbruggen B, Dardikh M, Polenewen R, van Duren C, Meijer P (2011) The factor VIII inhibitor assays can be standardized: results of a workshop. J Thromb Haemost 9: 2003–2008. 21854536 10.1111/j.1538-7836.2011.04479.x

[9] Jacquemin M, Desqueper B, Benhida A, vander Elst L, Hoylaerts M, et al (1998) Mechanism and kinetics of factor VIII inactivation: study with an IgG4 monoclonal antibody derived from a hemophilia A patient with inhibitor. Blood 92: 496–506. 9657749

[10] Gilles JGG, Grailly SC, De Maeyer M, Jacquemin MG, VanderElst LP, et al (2004) In vivo neutralization of a C2 domain-specific human anti-Factor VIII inhibitor by an anti-idiotypic antibody. Blood 103: 2617–2623. 14670927 10.1182/blood-2003-07-2207

[11] Villard S, Lacroix-Desmazes S, Kieber-Emmons T, Piquer D, Grailly S, et al (2003) Peptide decoys selected by phage display block in vitro and in vivo activity of a human anti-FVIII inhibitor. Blood 102: 949–952. 12676786 10.1182/blood-2002-06-1886

[12] Forrer P, Stumpp MT, Binz HK, Plückthun A (2003) A novel strategy to design binding molecules harnessing the modular nature of repeat proteins. FEBS letters 539: 2–6. 12650916 10.1016/s0014-5793(03)00177-7

[13] Zahnd C, Wyler E, Schwenk JM, Steiner D, Lawrence MC, et al (2007) A designed ankyrin repeat protein evolved to picomolar affinity to Her2. Journal of molecular biology 369: 1015–1028. 17466328 10.1016/j.jmb.2007.03.028

[14] Baumann MJ, Eggel A, Amstutz P, Stadler BM, Vogel M (2010) DARPins against a functional IgE epitope. Immunology letters 133: 78–84. 20673836 10.1016/j.imlet.2010.07.005

[15] Boersma YL, Chao G, Steiner D, Wittrup KD, Pluckthun A (2011) Bispecific designed ankyrin repeat proteins (DARPins) targeting epidermal growth factor receptor inhibit A431 cell proliferation and receptor recycling. Journal of Biological Chemistry 286: 41273–41285. 21979953 10.1074/jbc.M111.293266 PMC3308840

[16] Vogel M, Keller-Gautschi E, Baumann MJ, Amstutz P, Ruf C, et al (2007) Designed ankyrin repeat proteins as anti-idiotypic-binding molecules. Annals of the New York Academy of Sciences 1109: 9–18. 17785285 10.1196/annals.1398.002

[17] Binz HK, Stumpp MT, Forrer P, Amstutz P, Plückthun A (2003) Designing repeat proteins: well-expressed, soluble and stable proteins from combinatorial libraries of consensus ankyrin repeat proteins. Journal of molecular biology 332: 489–503. 12948497 10.1016/s0022-2836(03)00896-9

[18] Amstutz P, Binz H, Zahnd C (2006) Ribosome display: in vitro selection of protein–protein interactions: Cell Biology-A Laboratory Handbook.

[19] Hanes J, Pluckthun A (1997) In vitro selection and evolution of functional proteins by using ribosome display. Proc Natl Acad Sci U S A 94: 4937–4942. 9144168 10.1073/pnas.94.10.4937 PMC24609

[20] Zahnd C, Amstutz P, Plückthun A (2007) Ribosome display: selecting and evolving proteins in vitro that specifically bind to a target. Nature methods 4: 269–279. 17327848 10.1038/nmeth1003

[21] Binz HK, Amstutz P, Kohl A, Stumpp MT, Briand C, et al (2004) High-affinity binders selected from designed ankyrin repeat protein libraries. Nature biotechnology 22: 575–582. 10.1038/nbt962 15097997

[22] Eggel A, Baumann MJ, Amstutz P, Stadler BM, Vogel M (2009) DARPins as bispecific receptor antagonists analyzed for immunoglobulin E receptor blockage. Journal of molecular biology 393: 598–607. 19683003 10.1016/j.jmb.2009.08.014

[23] Lavigne-Lissalde G, Tarrade C, Lapalud P, Chtourou S, Schved J, et al (2008) Simultaneous detection and epitope mapping of anti-factor VIII antibodies. Thromb Haemost 99: 1090–1096. 18521513 10.1160/TH07-08-0497

[24] Moreau A, Lacroix-Desmazes S, Stieltjes N, Saenko E, Kaveri SV, et al (2000) Antibodies to the FVIII light chain that neutralize FVIII procoagulant activity are present in plasma of nonresponder patients with severe hemophilia A and in normal polyclonal human IgG. Blood 95: 3435–3441. 10828026

[25] Kopecky E-M, Greinstetter S, Pabinger I, Buchacher A, Römisch J, et al (2006) Mapping of FVIII inhibitor epitopes using cellulose-bound synthetic peptide arrays. J Immunol Methods 308: 90–100. 16376372 10.1016/j.jim.2005.10.016

[26] Spiegel P, Jacquemin M, Saint-Remy J, Stoddard B, Pratt K (2001) Structure of a factor VIII C2 domain-immunoglobulin G4kappa Fab complex: identification of an inhibitory antibody epitope on the surface of factor VIII. Blood 98: 13–19. 11418455 10.1182/blood.v98.1.13

