# Supplementary figures and images for: Designed Ankyrin Repeat Proteins: A New Approach to Mimic Complex Antigens for Diagnostic Purposes?
Source: PLoS One. 2013 Apr 23;8(4):e60688. doi: 10.1371/journal.pone.0060688 (PMC3634029; doi:10.1371/journal.pone.0060688)

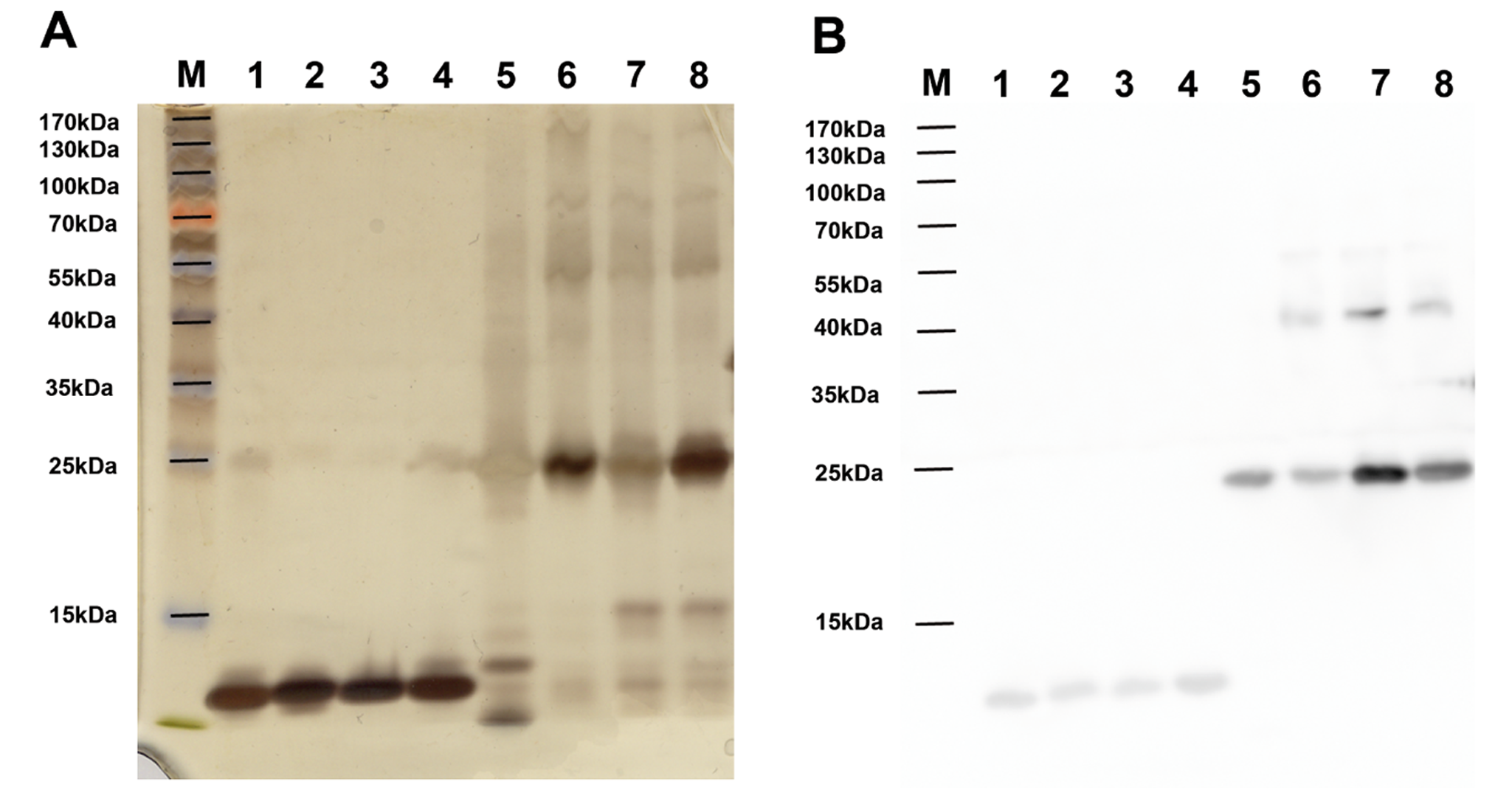

Supplement: Figure S1 — Purity and integrity of DARPins analyzed by SDS PAGE and Westernblot. Monovalent and divalent DARPins used in this study were loaded at 30pmoles per lane on a 12% acrylamide gel. Proteins were stained with silver ions (A) or blotted to a nitrocellulose membrane, stained with a murine anti-His6 antibody (Qiagen) followed by a peroxidase labeled anti-mouse IgG antibody (Jackson ImmunoResearch) and developed with chemiluminescence (B). 1, eBo01; 2, eBo38; 3, eBo89; 4, eBo90; 5, eBo01-01, 6, eBo01-38; 7, eBo38-01; 8, eBo38-38. Staining of eBo01-01 with silver ions bleached, the presence of protein was confirmed with coomassie-staining (not shown). A small degree of polymerization of both monomeric and dimeric DARPins is observed, as is usually the case. Also some degradation is visible and probably a small contamination of dimeric DARPins with monomeric ones (lines 6–8). (TIF) [file pone.0060688.s001.tif]
